# Post 90-day outcomes of acute ischemic stroke patients following thrombectomy: analysis of real-world data

**DOI:** 10.3389/fneur.2025.1543101

**Published:** 2025-04-30

**Authors:** Adnan I. Qureshi, William I. Baskett, Ibrahim A. Bhatti, Bruce Ovbiagele, Farhan Siddiq, Daniel E. Ford, Camilo R. Gomez, Daniel F. Hanley, Chi-Ren Shyu

**Affiliations:** ^1^Zeenat Qureshi Stroke Institutes, Columbia, MO, United States; ^2^Department of Neurology, University of Missouri, Columbia, MO, United States; ^3^Department of Data Science and Informatics, University of Missouri, Columbia, MO, United States; ^4^Department of Neurosurgery, University of Missouri, Columbia, MO, United States; ^5^Department of Neurology, University of California, San Francisco, San Francisco, CA, United States; ^6^Johns Hopkins University, Baltimore, MD, United States; ^7^Department of Electrical Engineering and Computer Science, University of Missouri, Columbia, MO, United States

**Keywords:** ischemic stroke, thrombectomy, long-term outcomes, survival rates, real-world data

## Abstract

**Background:**

Previous studies have focused on 90-day outcomes in acute ischemic stroke patients who undergo thrombectomy, although long-term outcomes are not well understood. We compared the long-term rates of survival and new stroke recurrence among acute ischemic stroke patients who did and did not undergo thrombectomy.

**Methods:**

Using the Oracle Real-World Data (a de-identified large data source of multicenter electronic health records covering the period of January 2016 to January 2023), we analyzed 3,934 acute ischemic stroke patients who underwent thrombectomy and 3,934 propensity-matched controls of acute ischemic stroke patients who did not undergo thrombectomy. The risk of death or palliative care and new stroke following >90 days post-admission was ascertained using Cox proportional hazards regression analysis to adjust for potential confounders. We also estimated the rate of new stroke and palliative care-free survival using Kaplan–Meier survival analysis.

**Results:**

Among 3,934 acute ischemic stroke patients who underwent thrombectomy, 2,660 patients either died or received palliative care or developed new stroke (median follow-up period of 775 days post-initial stroke admission; interquartile range Q1 = 356 days, Q3 = 1,341 days). The 2-year new stroke and palliative care-free survival were 36.6 and 45.8% among patients who did and did not undergo thrombectomy, respectively (adjusted hazard ratio [HR], 1.19, 95% confidence interval [CI], 1.12–1.26). The risk of palliative care or death was not different (adjusted HR, 0.89, 95% CI, 0.77–1.02) between both groups, but the risk of new stroke was higher among patients who underwent thrombectomy (adjusted HR, 1.25, 95% CI, 1.18–1.33).

**Conclusion:**

Acute ischemic stroke patients who undergo thrombectomy are at greater risk of new stroke, palliative care, or death after 90 days, primarily driven by the occurrence of stroke. There is a need for closer surveillance and enhanced recurrent stroke prevention in this high-risk group.

## Introduction

Randomized clinical trials evaluating thrombectomy have used 90-day post-procedure outcomes as the primary endpoint (1). Few randomized clinical trials have ascertained outcomes beyond 90 days (2). Previous single-center studies, based on data from acute ischemic stroke patients treated with thrombectomy outside of clinical trials, have reported relatively poor survival over 1 to 5 years post-procedure (3–6). Low real-world survival rates have been attributed to factors such as advanced age, high severity of neurologic deficits, pre-existing disability, and medical comorbidities of patients treated outside of clinical trials (7). In addition, the available national representative real-world data analyses are based on populations from Germany and China (4, 6). In an analysis of 18,506 patients with acute ischemic stroke treated with thrombectomy registered in any of the 16 regional health insurances in Germany, the 1-year mortality in patients aged over 80 years was 55.4% among those treated with thrombectomy and 19.3% in the general population > 80 years of age (4). In an analysis of 657 patients with acute ischemic stroke treated with thrombectomy in the observational nationwide registry at 28 comprehensive stroke centers in China (6), 48.2% of the patients had died, and 28.2% had stroke recurrence within 5 years post-treatment. These results may not apply to the United States (US), especially in regions of low population density, medically underserved, or with an excessive proportion at an increased risk of cardiovascular disease (e.g., the “stroke belt”) (8). Results of long-term outcomes in acute ischemic stroke patients who undergo thrombectomy in the US are important from a patient perspective in regard to the quality of life years gained and economic analysis (9–11). Our study objective analyzed rates of incident stroke and survival after thrombectomy in acute ischemic stroke patients using a large cohort representative of the US.

## Methods

### Patients

We analyzed the Oracle Real-World Data extracted from the electronic medical records of health care facilities that have a data use agreement with Oracle Corporation using the Oracle Real-World Data January 2023. Data were contributed from 139 Oracle Real-World Data health systems that had records from approximately 1.4 billion medical encounters (12–15). The analysis used electronic medical records from encounters from January 2016 to January 2023. As part of the de-identification procedure, the dataset does not provide an identifier for the medical institution of a patient’s data or its precise location. The healthcare networks with Oracle are classified as integrated delivery networks/regional health authorities (*n* = 25), regional hospitals (*n* = 28), community healthcare (*n* = 60), academic hospitals (*n* = 11), and specialty hospitals (*n* = 15). The distribution of hospitals according to the first digit of the zip code of location is as follows: 6 (*n* = 41), 9 (*n* = 15), 5 (*n* = 17), 7 (*n* = 11), 2 (*n* = 7), 3 (*n* = 11), 8 (*n* = 12), 4 (*n* = 11), 0 (*n* = 9), and 1 (*n* = 5). We used the first digit of the zip code to provide the most granular estimate of location within the data. Participating hospitals are somewhat disproportionately located in the Midwest and Pacific West Areas.

### Patient selection

We used the *International Classification of Diseases, Tenth Revision, Clinical Modification* (*ICD-10-CM*) primary diagnosis codes I63, I65, and I66 to identify adult patients aged 18 years or greater admitted with acute ischemic stroke after 1 January 2016. The hospital admission with acute ischemic stroke was designated as the reference encounter. Furthermore, (1) patients had to have at least two medical encounters before their reference encounter and (2) patients had to have at least two encounters more than 90 days after the reference encounter admission. The requirement of ≥2 encounter years was intended to ensure that patients had an up-to-date prior medical history. The requirement for ≥2 subsequent encounters following the reference encounter was intended to ensure patients had follow-up data.

### Case–control design

We used ICD-10 procedure codes to identify patients who underwent thrombectomy (03CG3ZZ, 03CG3Z7, 03CH3Z7, 03CJ0ZZ, 03CJ3ZZ, 03CK3Z7, 03CK3ZZ, 03CL3Z7, 03CL3ZZ, 03CL0ZZ, 03CP3ZZ, 03CY3ZZ, and 00C73ZZ). We identified control patients from a sample of acute ischemic stroke patients admitted to the hospital who did not undergo thrombectomy. Each patient who underwent thrombectomy was matched with a patient who did not undergo thrombectomy using propensity score matching to reduce selection bias in the population since the selection of patients who did and did not undergo thrombectomy was not random, and this bias could affect the results. Propensity score matching reduces this bias by using a model to predict the probability that a given patient will receive a thrombectomy and then matching patients based on their predicted probabilities of being treated. Thrombectomy patients were matched with non-thrombectomy patients who had a similar propensity to receive thrombectomy. The model used to estimate propensity score utilized demographics, diagnoses from the Elixhauser comorbidity index prior to or during the index encounter, and diagnoses of stroke-related impairment during the index encounter.

Propensity score matching reduces but does not eliminate differences between populations. This step was undertaken primarily to eliminate large population differences prior to a more detailed analysis. All covariates in our analysis were binary. The post-matching of the distribution of covariate frequencies between patients who were treated with thrombectomy and matched controls, along with the statistical significance of the remaining differences, is provided in Supplementary Table 1. The relative differences in frequencies between the matched populations are visualized in Supplementary Figure 1. The matching process results in highly similar populations, though some statistically significant differences in covariate frequency remain. This is expected when a propensity score matches two dissimilar populations using a large number of covariates. Propensity score matching using a linear model minimizes aggregate differences between the two matched populations in a linear context. It is not unusual for some covariates to retain statistically significant differences between the populations even after matching. Covariates that are most likely to retain statistically significant differences between matched populations are those that are not strongly predictive of treatment despite occurring at different frequencies between treatment groups. These are variables where the independent predictive power when predicting treatment groups largely disappears when adjusted for the other covariates.

### Identification of incident stroke events and deaths

We used the ICD-10-CM diagnosis codes I63, I65, and I66 for acute ischemic stroke, I61 for intracerebral hemorrhage (ICH), and I60 for subarachnoid hemorrhage (SAH) to identify patients diagnosed with incident stroke events. We ascertained deaths from any cause through patient discharge records. Palliative care was identified at any encounter with the ICD-10-CM diagnosis code Z51.5.

### Ascertainment of data

Factors that affect the risk of stroke events and survival as identified in previous studies (16–18) were extracted for each patient at any encounter using *ICD-10-CM* codes for hypertension (I10, O10.0, O10.9, I16, and I67.4), diabetes mellitus (E08, E09, E10, E11, and E13), hyperlipidemia (E78), nicotine dependence/tobacco use (F17), alcohol use or abuse (F10), atrial fibrillation (I48), congestive heart failure (I09.81, I11.0, and I50), acute kidney injury (AKI) (N17), and peripheral vascular disease (I70, I71, I72.0, I72.1, I72.2, I72.3, I72.4, I72.8, I72.9, I73.1, I73.8, I73.9, I74.2, I74.3, I74.4, I76, I77.1, I77.71, I77.72, I77.73, I77.74, I77.79, I79, K55.1, K55.8, K55.9, and Z95.82), acute MI(I21), or stroke (acute ischemic stroke [I63.9] ICH[161.9], or SAH[160.9]) using afore mentioned codes as shown in Supplementary Table 2. We used the following neurological deficits using ICD-10 diagnosis codes: aphasia (I69.320, I69.920, R47.01), hemiplegia (I69.35, G81), neglect (R41.4), somnolence, stupor, and coma (R.40), dysphagia (R13.1), and homonymous hemianopsia (H53.46) as indicators of neurological severity during index hospitalization (12, 13). Post-procedure ICH was defined by ICD-10-CM codes of either ICH or SAH during a reference encounter for acute ischemic stroke and thrombectomy. We used ICD diagnosis code R29.7 to identify the National Institutes of Health Stroke Scale (NIHSS) score, further categorized in strata as follows: R29.70-NIHSS score 0–9; R29.71-NIHSS score 10–19; R29.72-NIHSS score 20–29; R29.73-NIHSS score 30–39; and R29.74-NIHSS score 40–42. The discharge status at the reference encounter for acute ischemic stroke was classified as routine (discharge home) or non-routine (destination other than home).

### Statistical analysis

We calculated the proportion of patients who had incident stroke events and deaths with a 95% confidence interval (CI) without continuity correction. We performed Cox proportional hazards analysis including all cases and controls to identify the independent effect of thrombectomy on the composite endpoint of new stroke, palliative care, or death and included age, sex, race, and other acute and chronic conditions diagnosed prior to or during the reference encounter. All the hypothesis tests were two-sided, with a *p*-value of <0.05 considered statistically significant, and all the analyses were conducted using R (version 3.6.1). We also estimated the rate of new stroke and palliative care-free survival using Kaplan–Meier survival analysis. The baseline National Institutes of Health Stroke Scale Score (NIHSS) score at index hospitalization was not available for all patients. To assess the impact of the NIHSS score on the association between thrombectomy and composite endpoint, we performed a sensitivity analysis, which included only patients with baseline NIHSS scores available in the model and compared the association between thrombectomy and composite endpoint after adjustment for NIHSS score with the association in the overall model without NIHSS scores. We performed another sensitivity analysis using a Fine-Gray competing risk regression analysis to confirm the results of the Cox proportional hazards analysis.

## Results

### Overall rates of the incident composite endpoint

**Figure 1:**
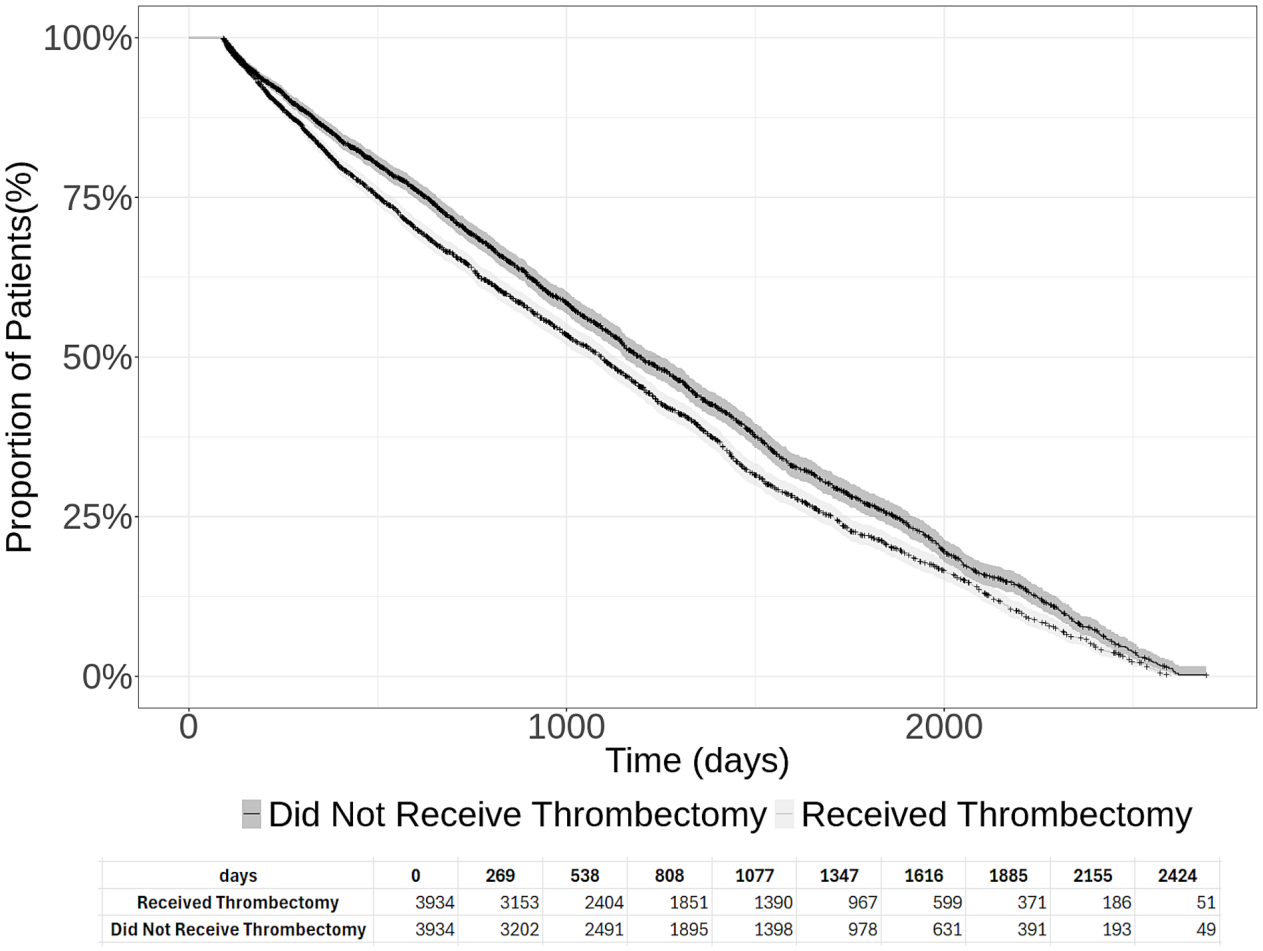
Cumulative new stroke and palliative care-free survival in acute ischemic stroke patients who underwent thrombectomy and those who did not undergo thrombectomy. The 95% confidence intervals are provided, and the number at risk at each time interval is presented at the bottom of the figure.

Among 3,934 acute ischemic stroke patients who underwent thrombectomy, 2,660 patients died, received palliative care, or developed new stroke (median follow-up period of 749 days post-initial stroke admission; interquartile range Q1 = 332 days, Q3 = 1,339 days). Table 1 presents the event rates of new stroke, palliative care, and death among patients who did and did not undergo thrombectomy. The rate of new stroke was higher among thrombectomy patients (63.3%) compared to non-thrombectomy patients (53.1%). Palliative care was slightly lower in the thrombectomy group (8.4% vs. 9.9%), and mortality rates were similar between groups (6.4% vs. 6.5%). The overall composite endpoint occurred in 67.6% of thrombectomy patients compared to 59.4% of non-thrombectomy patients. Among 3,934 acute ischemic stroke patients who did not undergo thrombectomy, 2,339 patients either died or received palliative care or developed new stroke (median follow-up period of 775 days post-initial stroke admission; interquartile range Q1 = 356 days, Q3 = 1,341 days) (see Figure 1). The 2-year new stroke and palliative care-free survival were 36.6 and 45.8% among patients who did and did not undergo thrombectomy, respectively (log-rank test *p* = 2×10^-8).

**Table 1:** Event rate of new stroke, palliative care, and/or death in acute ischemic stroke patients according to whether thrombectomy was performed.

Outcomes	Patients who underwent thrombectomy *N* (%)	Patients who did not undergo thrombectomy *N* (%)
New stroke	2,490 (63.3)	2087 (53.1)
Palliative care	330 (8.4)	391 (9.9)
Death	253 (6.4)	256 (6.5)
Composite endpoint	2,660 (67.6)	2,339 (59.4)

### Patients who underwent thrombectomy and risk of the composite endpoint

The risk of new stroke, palliative care, or death was significantly higher in acute ischemic stroke patients who underwent thrombectomy compared with those who did not undergo thrombectomy (adjusted hazard ratio [HR], 1.19, 95% CI, 1.12–1.26) after adjustment for potential confounders (Table 2). Overall risk factors for the composite endpoint in the entire cohort were age >80 years (HR 1.20); occurrence of post-procedure ICH (adjusted HR 1.23), post-procedure aphasia (adjusted HR 1.18), non-routine discharge (adjusted HR 1.14), and comorbidities including thrombosis (adjusted HR 1.37), metastatic solid tumors (adjusted HR 2.04), congestive heart failure (adjusted HR 1.09), peripheral vascular disease (adjusted HR 1.08), deficiency anemia (adjusted HR 1.11), and diagnosis of paralysis (adjusted HR 1.30) during follow-up visits (Table 3).

**Table 2:** Predictors of new stroke, palliative care, and/or death in acute ischemic stroke patients according to whether thrombectomy was performed.

	Patients who underwent thrombectomy		Patients who did not undergo thrombectomy	
	Hazard ratio	95% confidence interval	Hazard ratio	95% confidence interval
Age<50 years	Reference	Reference	Reference	Reference
Age 50–59 years	0.91	0.77–1.09	1.19	0.95–1.49
Age 60–69 years	0.95	0.81–1.12	1.26	1.03–1.54
Age 70–79 years	0.88	0.75–1.03	1.12	0.92–1.38
Age>80 years	0.99	0.83–1.18	1.54	1.24–1.93
Post-procedure ICH	1.38	1.17–1.61	1.04	0.84–1.28
Post-procedure aphasia	1.12	0.97–1.30	1.20	1.03–1.39
Diagnosis of thrombosis	1.80	1.14–2.84	1.17	0.78–1.77
Diagnosis of deficiency anemia	1.07	0.96–1.20	1.14	1.01–1.28
Diagnosis of rheumatoid arthritis and collagen vascular disorders	1.31	0.88–1.94	1.79	1.18–2.72
Metastatic solid tumors	2.78	1.94–3.99	1.52	1.02–2.26
Diagnosis of paralysis	1.20	0.87–1.64	1.24	0.97–1.59
Peripheral vascular disease	1.02	0.93–1.12	1.13	1.02–1.26
Congestive heart failure	1.04	0.93–1.17	1.18	1.04–1.34
Non-routine discharge	1.28	1.15–1.44	1.19	1.07–1.32

**Table 3:** Overall predictors of new stroke, palliative care, and/or death in acute ischemic stroke patients.

Events	Proportion of patients matching criteria with the composite endpoint	Proportion of patients matching criteria without the composite endpoint	Hazard ratio	95% Confidence interval
Patients receiving thrombectomy	2,660 (53%)	1,274 (44%)	1.19	1.12–1.26
Age<50 years	370 (7%)	230 (8%)	Reference	Reference
Age 50–59 years	613 (12%)	349 (12%)	0.99	0.86–1.13
Age 60–69 years	1,408 (28%)	724 (25%)	1.04	0.92–1.18
Age 70–79 years	1,609 (32%)	932 (32%)	0.96	0.85–1.09
Age>80 years	881 (18%)	546 (20%)	1.2	1.05–1.37
Post-procedure ICH	316 (6%)	163 (6%)	1.23	1.09–1.40
Post-procedure aphasia	1,495 (29%)	752 (26%)	1.18	1.07–1.31
Diagnosis of thrombosis	161 (1%)	91 (1%)	1.37	1.01–1.85
Diagnosis of deficiency anemia	1,034 (21%)	626 (22%)	1.11	1.03–1.20
Diagnosis of rheumatoid arthritis and collagen vascular disorders	193 (4%)	109 (4%)	1.49	1.13–1.98
Metastatic solid tumors	70 (1%)	30 (1%)	2.04	1.57–2.65
Diagnosis of paralysis	2,301 (46%)	1,206 (42%)	1.30	1.11–1.53
Peripheral vascular disease	1,257 (25%)	673 (23%)	1.08	1.01–1.16
Congestive heart failure	930 (19%)	553 (19%)	1.09	1.01–1.19
Non-routine discharge	2,352 (51%)	1,330 (46%)	1.14	1.06–1.23

### Patients who underwent thrombectomy and risk of various endpoints

The risk of palliative care or death was not significantly different (adjusted HR, 0.89, 95% CI, 0.77–1.02), but the risk of new stroke was significantly higher (adjusted HR, 1.25, 95% CI, 1.18–1.33) among patients who underwent thrombectomy.

### Risk factors for the composite endpoint among patients who underwent thrombectomy

Among patients who underwent thrombectomy, post-procedure ICH (adjusted HR 1.38), metastatic solid tumors (adjusted HR 2.78), diagnosis of paralysis (adjusted HR 1.20), prior diagnosis of thrombosis (adjusted HR 1.80), and patients with non-routine discharge were at higher risk for the composite endpoint than patients who did not undergo thrombectomy. In this group, patients of age >80 years (adjusted HR 1.44), those with a previous acute diagnosis of rheumatoid arthritis (adjusted HR 1.79), peripheral vascular disease (adjusted HR 1.13), congestive heart failure (adjusted HR 1.18), diagnosis of paralysis (adjusted HR 1.24), and deficiency anemia (adjusted HR 1.14) were at higher risk for the composite endpoint than patients who underwent thrombectomy.

### Sensitivity analysis

For the subset of 2,929 patients who had recorded baseline NIHSS scores, the HR for the composite endpoint associated with thrombectomy was not different when the NIHSS scores were included in the model (adjusted HR, 1.24, 95% CI, 1.12–1.37) compared to when the NIHSS score was not added (adjusted HR, 1.23, 95% CI, 1.12–1.36). The results derived using the Fine-Gray competing risk analysis were similar to the Cox proportional hazards analysis. The risk of new stroke, palliative care, or death was significantly higher in acute ischemic stroke patients who underwent thrombectomy compared with those who did not undergo thrombectomy (adjusted HR 1.19, 95% CI 1.13–1.25, *p* < 0.0001) after adjustment for potential confounders.

## Discussion

Our results demonstrate that acute ischemic stroke patients who undergo thrombectomy constitute a high-risk group that remains at high risk of recurrent stroke, palliative care, and death after the first 90 days. This risk was higher in patients who required thrombectomy compared with patients with other characteristics also associated with the composite endpoint. The risk factors for the composite endpoint were different in patients who underwent thrombectomy and those who did not. Our results also highlight the importance of ascertaining post-90-day outcomes since studies demonstrating the efficacy of thrombectomy have focused on outcomes within 90 days post-procedure and, by design, do not allow accurate forecasting of long-term benefits (1). It is important to recognize that a higher rate of the composite endpoint is not related to thrombectomy but to the characteristics of patients who require thrombectomy. In particular, the control patients have a greater proportion of patients with lacunar infarction, which may have higher survival in the first year compared with other stroke subtypes (19–21). Furthermore, acute ischemic stroke patients who require thrombectomy may have a higher burden of underlying cardiovascular diseases (22, 23).

The mortality rates ranged from 28.5 to 78.0% over 1–5 years in acute ischemic stroke patients after undergoing thrombectomy in single-center studies (3, 5, 24–27). The 1–2 year mortality rate was 26 and 31% for patients treated with thrombectomy and best medical treatment in a combined analysis of 1,362 patients in clinical trials (2). The 1-year mortality in patients aged over 80 years was 55.4% compared with 28.5% in patients who were aged ≤80 years among those treated with thrombectomy and 19.3% in the general population > 80 years of age in an analysis of 18,506 patients with acute ischemic stroke treated with thrombectomy in the Allgemeine Ortskrankenkasse, a consortium of 16 regional health insurances in Germany (4). The 1-year rate of moderate to severe disability was 35.5% in patients aged > 80 years compared with 33.2% of patients who were aged ≤80 years. Only 38.4% of patients aged > 80 years were living at home compared with 62.8% in patients who were aged ≤80 years. Hypertension, dementia, atrial fibrillation, and flutter were associated with a higher likelihood of moderate to severe disability. Gong et al. (6) reported 657 patients with acute ischemic stroke treated with thrombectomy in the observational nationwide registry at 28 comprehensive stroke centers in China. At 5 years, 48.2% (23.5% died after 90 days) of the patients had died, and 28.2% had stroke recurrence. Younger age, lower disability at 90 days, and absence of stroke recurrence were significantly associated with functional independence at 5 years. Advanced age, higher disability at 90 days, and atrial fibrillation were associated with stroke recurrence.

There has been an emphasis on the identification of patients with cardiovascular diseases who are at high risk for subsequent cardiovascular events and death (28–30) since there has been no change in stroke-free survival in the last decade among stroke survivors (31). Patients undergoing thrombectomy are most likely to benefit from targeted secondary prevention strategies and the extended care model.

The American Heart Association/American Stroke Association (AHA/ASA) 2021 (32) recommends the management of vascular risk factors, lifestyle factors, changing patient behaviors, antithrombotic therapy, and specific strategies for atrial fibrillation and extracranial carotid artery disease. Schwalm et al. (33) identified the inadequacy of secondary prevention despite recommendations in AHA/ASA guidelines because of multiple barriers at the patient (knowledge and access and costs), clinician (knowledge, attitude, limited time, and limited resources), and health system (fragmentation of care, low priority setting, and limited infrastructure) level. Self-management by the patient (34) and extended care by a nurse practitioner/physician assistant care manager (35, 36) assist in compliance with prescribed strategies. The Prevent Recurrence of All Inner-city Strokes through Education (PRAISE) trial (37) and Boden-Albala et al. (36) found that community-based self-management increased the proportion of patients with controlled blood pressure at 6 months.

Lennon et al. (38) performed a meta-analysis of 16 randomized trials involving 2,478 patients. Interventions incorporating any key component of health education/promotion/counseling on lifestyle and/or aerobic exercise (compared to usual care ± a sham) intervention increased physical activity participation and reduced blood pressure in patients with ischemic stroke or transient ischemic attack (TIA). Deijle et al. (39) performed a meta-analysis of 22 trials (*n* = 2,574 patients) that either used a behavior change intervention (*n* = 2 trials), cardiovascular fitness intervention (*n* = 5 trials), or a combined intervention (*n* = 5 trials). There was a significant reduction in systolic blood pressure due to the lifestyle interventions applied, compared with usual care. No significant effect was found on cardiovascular events, mortality, diastolic blood pressure, or cholesterol. In the subgroup analyses, trials with cardiovascular fitness interventions, trials with an intervention that lasted longer than 4 months, and interventions that used >3 behavior change techniques were more effective in reducing systolic blood pressure. (40).

### Limitations

We relied on Oracle Real-World Data, which offers limited information regarding the time elapsed between symptom onset and hospital arrival, the severity of neurological impairments, and the findings of diagnostic tests such as neuroimaging. The introduction of the International Classification of Diseases, 10th Revision (ICD-10) codes has permitted the coding of patients’ NIHSS scores in hospital discharge diagnosis codes (41, 42). The lack of NIHSS score in every patient included in our analysis pose a significant limitation since NIHSS scores have high predictive value for determining long term outcomes in acute ischemic stroke patients (43, 44) We repeated the analysis after adjusting for NIHSS scores (when available due to a high level of accuracy) and did not identify any change in the direction or strength of association between thrombectomy and the composite endpoint. There is a high concordance between the NIHSS score ascertained by ICD-10-CM codes and NIHSS scores documented in medical records with an intraclass correlation coefficient of 0.93 (45). Less than 10% of patients have a large discordance between NIHSS scores ascertained by ICD-10-CM codes and NIHSS scores documented in the registry (41). Furthermore, the specific reasons for excluding patients from receiving intravenous (IV) thrombolytic therapy or thrombectomy were not documented in the dataset (13). Certain variables, such as ICH and pneumonia, are not characterized by severity. Additionally, we used ICD-10 codes to identify new-onset cardiovascular events as used in previous studies (46, 47). ICD-10 diagnosis codes have a high positive predictive value to identify acute ischemic stroke from the principle discharge diagnosis (48). A review of 77 studies published from 1976 to 2015 (49) reported that the sensitivity of ICD-9 or ICD-10 codes for any cerebrovascular disease was ≥ 82% in majority of the studies, and specificity and negative predictive values were both ≥ 95%. The positive predictive values were ≥ 93% for SAH, 89% for SAH, and 82% for ischemic stroke (49). Lawrence et al. (50) reported very high sensitivity but moderate specificity for ICD-10 codes in identifying stroke events during follow-up in a cohort study. ICD-10-CM diagnosis code Z51.5 for palliative care is inclusive of hospice since the code is used when patients receive hospice services, including palliative and end-of-life care. The code does not consider that majority of the palliative care is not provided by hospice and palliative medicine specialists but rather by their primary care providers (51). The use of palliative care is highly suggestive of a high likelihood of severe disability categorized by the modified Rankin scale of 4 and 5 (52). However, our dataset lacks information that would be crucial for a medical indication of palliative care, such as symptom burden, prognosis, concurrent medical conditions, patients’ and families’ preferences, and psychosocial wellbeing (53). It would be preferable to use actual modified Rankin scale grades and define severe disability based on grades 5 and 4. The available data do not allow ascertaining functional outcomes using the modified Rankin scale at discharge or any of the subsequent encounters.

We used discharge destination, which has a high predictive value for predicting death and disability at 3 months as defined by the modified Rankin scale (54). We also used ICD-10 codes in follow-up encounters, such as those coded as dependence on a wheelchair or need for constant supervision to ascertain disability, which have not been validated yet. We acknowledge that the propensity score model to identify matching controls has limitations due to the inability to account for unmeasured confounders and biases due to imperfect matching. We used the Cox proportional hazards model for estimating the effect of thrombectomy on the hazard of the occurrence of the standard events used in the analysis of follow-up studies involving ischemic stroke patients. We acknowledge that an alternate approach would be using competing risk analysis (55) Competing risks arise in clinical research when there is more than one possible outcome during follow-up for survival data, and the occurrence of an outcome of interest can be precluded by another such as death. Using the Cox proportional hazards model may overestimate the incidence of events (compared with competing risk analysis), which was not the primary outcome of our analysis. Therefore, we performed a sensitivity analysis using the Fine-Gray competing risk analysis, and the results were similar to the Cox proportional hazards analysis.

We observed that the risk of new stroke, palliative care, or death after 90 days was significantly higher among acute ischemic stroke patients who underwent thrombectomy (compared with those who did not undergo thrombectomy), identifying these patients as a high-risk group that may benefit from long-term close surveillance and optimal implementation of evidence-based preventive interventions to maximize the benefit of thrombectomy.

## Data Availability

Publicly available datasets were analyzed in this study. This data can be found at: https://www.oracle.com/health/population-health/real-world-data/.
