# Supplementary material for: Post 90-day outcomes of acute ischemic stroke patients following thrombectomy: analysis of real-world data
Source: Front Neurol. 2025 Apr 30;16:1543101. doi: 10.3389/fneur.2025.1543101 (PMC12074927; doi:10.3389/fneur.2025.1543101)
Supplement: Supplementary file 1 [file Data_Sheet_1.docx]

| **Supplemental Table 1**: Variables used for propensity matching and frequencies post-matching in matched groups. p values calculated using fishers exact test |  |  |  |  |
| --- | --- | --- | --- | --- |
| (Immediate = index encounter, Previous = prior to index encounter) |  |  |  |  |
|  |  |  |  |  |
| **variable** | | **Received Thrombectomy** | **Did Not Receive Thrombectomy** | **P Value** |
| **Sex:Female** | | 0.4563 | 0.4265 | 0.0084 |
| **Sex:Male** | | 0.5437 | 0.5735 | 0.0084 |
| **Age:50-59** | | 0.1256 | 0.119 | 0.3896 |
| **Age:60-69** | | 0.2636 | 0.2783 | 0.1482 |
| **Age:70-79** | | 0.3129 | 0.333 | 0.06 |
| **Age:< 50** | | 0.089 | 0.0635 | <0.0001 |
| **Age:> 80** | | 0.1833 | 0.184 | 0.9536 |
| **Race:Asian** | | 0.0168 | 0.0178 | 0.7954 |
| **Race:Black** | | 0.0874 | 0.0679 | 0.0014 |
| **Race:Hispanic** | | 0.0041 | 0.0058 | 0.3356 |
| **Race:Other** | | 0.0836 | 0.0737 | 0.1115 |
| **Race:White** | | 0.8081 | 0.8348 | 0.0022 |
| **Immediate acute kidney injury** | | 0.0961 | 0.1131 | 0.015 |
| **Previous history of TIA or cerebral infarction** | | 0.0513 | 0.0488 | 0.6418 |
| **Previous history of traumatic brain injury** | | 0.0018 | 0.0031 | 0.3587 |
| **Previous transient cerebral ischemic attack** | | 0.0203 | 0.0219 | 0.695 |
| **Immediate transient cerebral ischemic attack** | | 0.0648 | 0.0895 | <0.0001 |
| **Previous Thrombosis** | | 0.0079 | 0.0086 | 0.8035 |
| **Previous acute myocardial infarction** | | 0.0376 | 0.0384 | 0.9062 |
| **Previous subarachnoid hemorrhage** | | 0.0025 | 0.0038 | 0.4236 |
| **Immediate subarachnoid hemorrhage** | | 0.0308 | 0.0282 | 0.5487 |
| **Immediate Deep venous thrombosis** | | 0.0348 | 0.0292 | 0.1787 |
| **Previous intracerebral hemorrhage** | | 0.0015 | 0.0013 | 1 |
| **Immediate intracerebral hemorrhage** | | 0.0707 | 0.0511 | 0.0003 |
| **Previous pulmonary embolism** | | 0.0094 | 0.0074 | 0.387 |
| **Immediate pneumonia** | | 0.0384 | 0.0402 | 0.7277 |
| **Immediate urinary tract infection** | | 0.0811 | 0.0793 | 0.8033 |
| **Immediate aphasia** | | 0.2883 | 0.2829 | 0.6177 |
| **Immediate hemiplegia** | | 0.4263 | 0.3963 | 0.0073 |
| **Immediate neglect** | | 0.0501 | 0.0402 | 0.0389 |
| **Immediate Somnolence, stupor and coma** | | 0.0857 | 0.0811 | 0.4882 |
| **Immediate dysphagia** | | 0.1718 | 0.1657 | 0.4888 |
| **Immediate sequelae of cerebrovascular disease** | | 0.1202 | 0.1322 | 0.1183 |
| **Immediate homonymous hemianopsia** | | 0.0264 | 0.0214 | 0.1606 |
| **Immediate dependence on renal dialysis** | | 0.0092 | 0.0117 | 0.3178 |
| **Immediate dependence on supplemental oxygen** | | 0.0119 | 0.0153 | 0.2427 |
| **Immediate bed confinement** | | 0.0031 | 0.0023 | 0.6632 |
| **Immediate need for assistance with personal care** | | 0.0069 | 0.0051 | 0.3803 |
| **Immediate need for continuous supervision** | | 0.0018 | 0.0031 | 0.3587 |
| **Immediate difficulty in walking/abnormalities of Gait** | | 0.0262 | 0.0231 | 0.424 |
| **Immediate dependence on wheelchair** | | 0.0048 | 0.0056 | 0.7546 |
| **Immediate other reduced mobility** | | 0.0402 | 0.0366 | 0.4456 |
| **Immediate dependence on respirator** | | 0.0056 | 0.0046 | 0.635 |
| **Immediate dependence on other enabling machines and devices [walker]** | | 0.0025 | 0.0023 | 1 |
| **Participation in clinical trial** | | 0.0353 | 0.0292 | 0.1422 |
| **(Comorbidity) atrial fibrillation** | | 0.2829 | 0.2491 | 0.0008 |
| **(Comorbidity) Dementia** | | 0.0127 | 0.0145 | 0.5594 |
| **(Comorbidity) Hemiplegia/paraplegia** | | 0.4047 | 0.3732 | 0.0044 |
| **(Comorbidity) Myocardial infarction** | | 0.1261 | 0.1347 | 0.2692 |
| **(Comorbidity) Mild liver disease** | | 0.0112 | 0.0112 | 1 |
| **(Comorbidity) Moderate or severe liver disease** | | 0.0033 | 0.0043 | 0.5839 |
| **(Comorbidity) Moderate or severe renal disease** | | 0.017 | 0.0221 | 0.1218 |
| **(Comorbidity) HIV/AIDS** | | 0.0038 | 0.0033 | 0.8503 |
| **(Comorbidity) Malignancy (any type)** | | 0.1261 | 0.1317 | 0.4798 |
| **(Comorbidity) Metastatic solid tumor** | | 0.0132 | 0.0122 | 0.7629 |
| **(Comorbidity) Connective tissue disease** | | 0.0313 | 0.0259 | 0.176 |
| **(Comorbidity) Diabetes mellitus** | | 0.2974 | 0.3114 | 0.1858 |
| **(Comorbidity) Diabetes mellitus w/ end-organ damage** | | 0.1904 | 0.2125 | 0.0156 |
| **(Comorbidity) Paralysis** | | 0.4603 | 0.4311 | 0.0097 |
| **(Comorbidity) Other neurologic disorders** | | 0.3965 | 0.3813 | 0.1724 |
| **(Comorbidity) Alcohol abuse** | | 0.0557 | 0.0663 | 0.0534 |
| **(Comorbidity) Drug abuse** | | 0.0384 | 0.0399 | 0.7714 |
| **(Comorbidity) Psychoses** | | 0.0292 | 0.0302 | 0.8422 |
| **(Comorbidity) Depression** | | 0.1362 | 0.1383 | 0.8186 |
| **(Comorbidity) Congestive heart failure** | | 0.1952 | 0.1817 | 0.1339 |
| **(Comorbidity) Peripheral vascular disease** | | 0.2466 | 0.244 | 0.8136 |
| **(Comorbidity) Valvular Heart Disease** | | 0.1693 | 0.1457 | 0.0044 |
| **(Comorbidity) Hypertension** | | 0.7519 | 0.7895 | 0.0001 |
| **(Comorbidity) Chronic pulmonary disease** | | 0.2163 | 0.2224 | 0.5309 |
| **(Comorbidity) Peptic ulcer disease** | | 0.0168 | 0.0211 | 0.1855 |
| **(Comorbidity) Liver disease** | | 0.0112 | 0.0112 | 1 |
| **(Comorbidity) Renal Failure** | | 0.1601 | 0.1772 | 0.0469 |
| **(Comorbidity) Solid tumor without metastasis** | | 0.0663 | 0.0559 | 0.0597 |
| **(Comorbidity) Metastatic cancer** | | 0.0132 | 0.0122 | 0.7629 |
| **(Comorbidity) Lymphoma** | | 0.0076 | 0.0058 | 0.4085 |
| **(Comorbidity) Coagulopathy** | | 0.0689 | 0.0719 | 0.6279 |
| **(Comorbidity) Blood loss anemia** | | 0.0165 | 0.017 | 0.9301 |
| **(Comorbidity) Deficiency anemia** | | 0.2199 | 0.2021 | 0.0566 |
| **(Comorbidity) Rheumatoid arthritis and collagen vascular disorders** | | 0.0422 | 0.0346 | 0.0886 |
| **(Comorbidity) Diabetes mellitus (uncomplicated)** | | 0.285 | 0.3005 | 0.1371 |
| **(Comorbidity) Diabetes mellitus (complicated)** | | 0.1904 | 0.2125 | 0.0156 |
| **(Comorbidity) Hypothyroidism** | | 0.1553 | 0.1584 | 0.7331 |
| **(Comorbidity) Obesity** | | 0.213 | 0.198 | 0.1056 |
| **(Comorbidity) Weight loss** | | 0.0498 | 0.0496 | 1 |
| **Immediate Intubation/mechanical ventilation** | | 0.0541 | 0.0425 | 0.0179 |
| **Immediate tracheostomy** | | 0.0104 | 0.0125 | 0.4582 |
| **Immediate thrombolysis** | | 0.139 | 0.1416 | 0.7702 |
| **Immediate routine discharge** | | 0.6197 | 0.6772 | <0.0001 |
| **Immediate non-routine discharge (excluding death)** | | 0.4667 | 0.515 | <0.0001 |

**Supplemental Figure 1:** Visualization of post-matching covariate frequencies


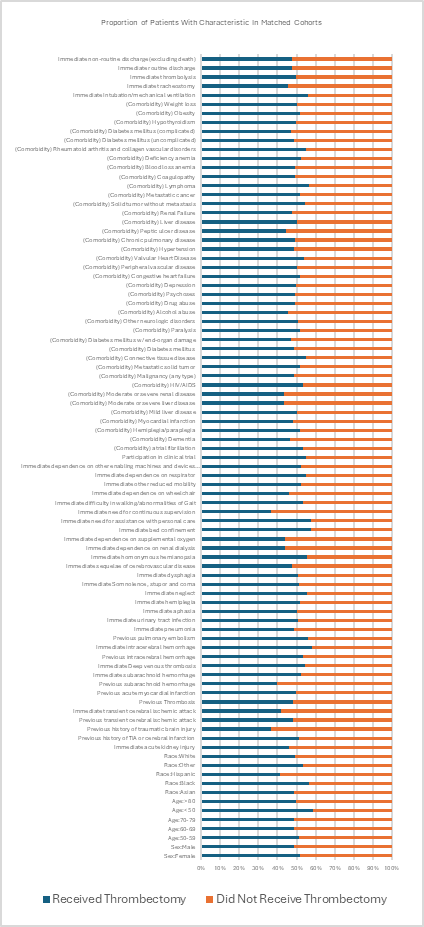

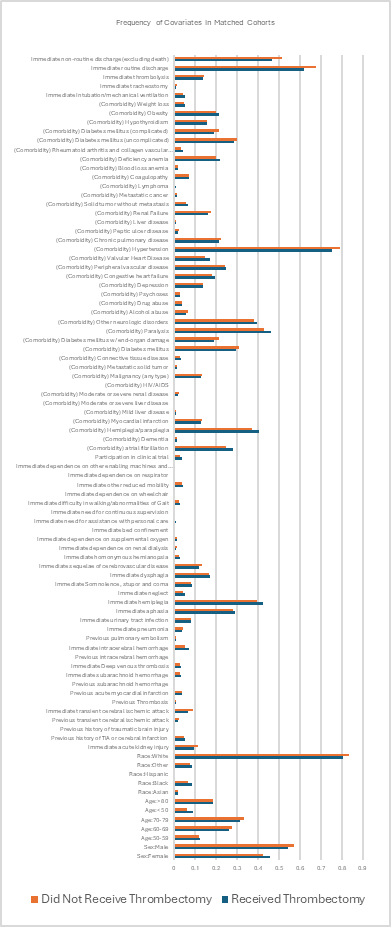


**Supplemental Table 2:** ICD-10 codes of comorbidities influencing stroke rate and survival outcomes

| Atrial fibrillation | I48 |
| --- | --- |
| Cerebrovascular disease | I60/I61/I62/I63/I64/I65/I66/I67.0/I67.1/I67.2/I67.4/I67.5/I67.6/I67.7/I67.8/I67.9/I68.1/I68.2/I68.8/I69/G45.0/G45.1/G45.2/G45.4/G45.8/G45.9/G46 |
| Dementia | F00/F01/F02/F051 |
| Hemiplegia/paraplegia | G81/G04.1/G82.0/G82.1/G82.2 |
| Mild liver disease | K70.2/K70.3/K73/K71.7/K74.0/K74.2/K74.6/K74.3/K74.4/K74.5 |
| Moderate or severe liver disease | K72.9/K76.6/K76.7/K72.1 |
| Moderate or severe renal disease | N18.6/Z99.2 |
| HIV/AIDS | B20/B21/B22/B23/B24 |
| Malignancy (any type) | Z85 |
| Metastatic solid tumor | C77/C78/C79/C80 |
| Connective tissue disease | M32/M34/M33.2/M05.3/M05.8/M05.9/M06.0/M06.3/M06.9/M05.0/M05.2/M05.1/M35.3 |
| Diabetes mellitus | E10.9/E11.9/E13.9/E14.9/E10.1/E11.1/E13.1/E14.1/E10.5/E11.5/E13.5/E14.5 |
| Diabetes mellitus w/ end-organ damage | E08.21/E08.22/E08.29/E08.311/E08.319/E08.321/E08.329/E08.331/E08.339/E08.341/E08.349/E08.351/E08.359/E08.36/E08.39/E08.40/E08.41/E08.42/E08.43/E08.44/E08.49/E08.51/E08.52/E08.59/E08.610/E08.618/E08.620/E08.621/E08.622/E08.628/E08.630/E08.638/E08.641/E08.649/E08.65/E08.69/E08.8/E09.21/E09.22/E09.29/E09.311/E09.319/E09.321/E09.329/E09.331/E09.339/E09.341/E09.349/E09.351/E09.359/E09.36/E09.39/E09.40/E09.41/E09.42/E09.43/E09.44/E09.49/E09.51/E09.52/E09.59/E09.610/E09.618/E09.620/E09.621/E09.622/E09.628/E09.630/E09.638/E09.641/E09.649/E09.65/E09.69/E09.8/E10.21/E10.22/E10.29/E10.311/E10.319/E10.321/E10.329/E10.331/E10.339/E10.341/E10.349/E10.351/E10.359/E10.36/E10.39/E10.40/E10.41/E10.42/E10.43/E10.44/E10.49/E10.51/E10.52/E10.59/E10.610/E10.618/E10.620/E10.621/E10.622/E10.628/E10.630/E10.638/E10.641/E10.649/E10.65/E10.69/E10.8/E11.21/E11.22/E11.29/E11.311/E11.319/E11.321/E11.329/E11.331/E11.339/E11.341/E11.349/E11.351/E11.359/E11.36/E11.39/E11.40/E11.41/E11.42/E11.43/E11.44/E11.49/E11.51/E11.52/E11.59/E11.610/E11.618/E11.620/E11.621/E11.622/E11.628/E11.630/E11.638/E11.641/E11.649/E11.65/E11.69/E11.8/E13.21/E13.22/E13.29/E13.311/E13.319/E13.321/E13.329/E13.331/E13.339/E13.341/E13.349/E13.351/E13.359/E13.36/E13.39/E13.40/E13.41/E13.42/E13.43/E13.44/E13.49/E13.51/E13.52/E13.59/E13.610/E13.618/E13.620/E13.621/E13.622/E13.628/E13.630/E13.638/E13.641/E13.649/E13.65/E13.69/E13.8/P70.2 |
| Paralysis | G04.1/G80.0/G80.1/G80.2/G80.4/G80.8/G80.9/G81.00/G81.01/G81.02/G81.03/G81.04/G81.10/G81.11/G81.12/G81.13/G81.14/G81.90/G81.91/G81.92/G81.93/G81.94/G82.20/G82.21/G82.22/G82.50/G82.51/G82.52/G82.53/G82.54/G83.0/G83.10/G83.11/G83.12/G83.13/G83.14/G83.20/G83.21/G83.22/G83.23/G83.24/G83.30/G83.31/G83.32/G83.33/G83.34/G83.4/G83.5/G83.81/G83.82/G83.83/G83.84/G83.89/G83.9/I69.031/I69.032/I69.033/I69.034/I69.039/I69.041/I69.042/I69.043/I69.044/I69.049/I69.051/I69.052/I69.053/I69.054/I69.059/I69.061/I69.062/I69.063/I69.064/I69.065/I69.069/I69.131/I69.132/I69.133/I69.134/I69.139/I69.141/I69.142/I69.143/I69.144/I69.149/I69.151/I69.152/I69.153/I69.154/I69.159/I69.161/I69.162/I69.163/I69.164/I69.165/I69.169/I69.231/I69.232/I69.233/I69.234/I69.239/I69.241/I69.242/I69.243/I69.244/I69.249/I69.251/I69.252/I69.253/I69.254/I69.259/I69.261/I69.262/I69.263/I69.264/I69.265/I69.269/I69.331/I69.332/I69.333/I69.334/I69.339/I69.341/I69.342/I69.343/I69.344/I69.349/I69.351/I69.352/I69.353/I69.354/I69.359/I69.361/I69.362/I69.363/I69.364/I69.365/I69.369/I69.831/I69.832/I69.833/I69.834/I69.839/I69.841/I69.842/I69.843/I69.844/I69.849/I69.851/I69.852/I69.853/I69.854/I69.859/I69.861/I69.862/I69.863/I69.864/I69.865/I69.869/I69.931/I69.932/I69.933/I69.934/I69.939/I69.941/I69.942/I69.943/I69.944/I69.949/I69.951/I69.952/I69.953/I69.954/I69.959/I69.961/I69.962/I69.963/I69.964/I69.965/I69.969/R532 |
| Other neurologic disorders | E75.00/E75.01/E75.02/E75.09/E75.10/E75.11/E75.19/E75.23/E75.25/E75.29/E75.4/F84.2/G10/G11.0/G11.1/G11.2/G11.3/G11.4/G11.8/G11.9/G12.0/G12.1/G12.20/G12.21/G12.22/G12.29/G12.8/G12.9/G13.2/G13.8/G20/G21.4/G24.01/G24.02/G24.09/G24.2/G24.8/G25.4/G25.5/G25.81/G30.0/G30.1/G30.8/G30.9/G31.01/G31.09/G31.1/G31.2/G31.81/G31.82/G31.83/G31.84/G31.85/G31.89/G31.9/G32.81/G35/G36.1/G36.8/G36.9/G37.0/G37.1/G37.2/G37.3/G37.4/G37.5/G37.8/G37.9/G40.001/G40.009/G40.011/G40.019/G40.101/G40.109/G40.111/G40.119/G40.201/G40.209/G40.211/G40.219/G40.301/G40.309/G40.311/G40.319/G40.401/G40.409/G40.411/G40.419/G40.501/G40.509/G40.801/G40.802/G40.803/G40.804/G40.811/G40.812/G40.813/G40.814/G40.821/G40.822/G40.823/G40.824/G40.89/G40.901/G40.909/G40.911/G40.919/G40.A01/G40.A09/G40.A11/G40.A19/G40.B01/G40.B09/G40.B11/G40.B19/G47.411/G47.419/G47.421/G47.429/G80.3/G89.0/G91.0/G91.1/G91.2/G91.3/G91.4/G91.8/G91.9/G93.7/G93.89/G93.9/G94/O99.350/O99.351/O99.352/O99.353/O99.354/O99.355/P91.60/P91.61/P91.62/P91.63/R41.0/R41.82/R47.01/R56.00/R56.01/R56.1/R56.9 |
| Alcohol abuse | F10.10/F10.120/F10.121/F10.129/F10.14/F10.150/F10.151/F10.159/F10.180/F10.181/F10.182/F10.188/F10.19/F10.20/F10.21/F10.220/F10.221/F10.229/F10.230/F10.231/F10.232/F10.239/F10.24/F10.250/F10.251/F10.259/F10.26/F10.27/F10.280/F10.281/F10.282/F10.288/F10.29/F10.921/F10.94/F10.950/F10.951/F10.959/F10.96/F10.97/F10.980/F10.981/F10.982/F10.988/F10.99 |
| Drug abuse | F11.10/F11.120/F11.121/F11.122/F11.129/F11.14/F11.150/F11.151/F11.159/F11.181/F11.182/F11.188/F11.19/F11.20/F11.21/F11.220/F11.221/F11.222/F11.229/F11.23/F11.24/F11.250/F11.251/F11.259/F11.281/F11.282/F11.288/F11.29/F12.10/F12.120/F12.121/F12.122/F12.129/F12.150/F12.151/F12.159/F12.180/F12.188/F12.19/F12.20/F12.21/F12.220/F12.221/F12.222/F12.229/F12.250/F12.251/F12.259/F12.280/F12.288/F12.29/F13.10/F13.120/F13.121/F13.129/F13.14/F13.150/F13.151/F13.159/F13.180/F13.181/F13.182/F13.188/F13.19/F13.20/F13.21/F13.220/F13.221/F13.229/F13.230/F13.231/F13.232/F13.239/F13.24/F13.250/F13.251/F13.259/F13.26/F13.27/F13.280/F13.281/F13.282/F13.288/F13.29/F14.10/F14.120/F14.121/F14.122/F14.129/F14.14/F14.150/F14.151/F14.159/F14.180/F14.181/F14.182/F14.188/F14.19/F14.20/F14.21/F14.220/F14.221/F14.222/F14.229/F14.23/F14.24/F14.250/F14.251/F14.259/F14.280/F14.281/F14.282/F14.288/F14.29/F15.10/F15.120/F15.121/F15.122/F15.129/F15.14/F15.150/F15.151/F15.159/F15.180/F15.181/F15.182/F15.188/F15.19/F15.20/F15.21/F15.220/F15.221/F15.222/F15.229/F15.23/F15.24/F15.250/F15.251/F15.259/F15.280/F15.281/F15.282/F15.288/F15.29/F16.10/F16.120/F16.121/F16.122/F16.129/F16.14/F16.150/F16.151/F16.159/F16.180/F16.183/F16.188/F16.19/F16.20/F16.21/F16.220/F16.221/F16.229/F16.24/F16.250/F16.251/F16.259/F16.280/F16.283/F16.288/F16.29/F18.10/F18.120/F18.121/F18.129/F18.14/F18.150/F18.151/F18.159/F18.17/F18.180/F18.188/F18.19/F18.20/F18.21/F18.220/F18.221/F18.229/F18.24/F18.250/F18.251/F18.259/F18.27/F18.280/F18.288/F18.29/F19.10/F19.120/F19.121/F19.122/F19.129/F19.14/F19.150/F19.151/F19.159/F19.16/F19.17/F19.180/F19.181/F19.182/F19.188/F19.19/F19.20/F19.21/F19.220/F19.221/F19.222/F19.229/F19.230/F19.231/F19.232/F19.239/F19.24/F19.250/F19.251/F19.259/F19.26/F19.27/F19.280/F19.281/F19.282/F19.288/F19.29/F55.0/F55.1/F55.2/F55.3/F55.4/F55.8/O99.320/O99.321/O99.322/O99.323/O99.324/O99.325 |
| Psychoses | F20.0/F20.1/F20.2/F20.3/F20.5/F20.81/F20.89/F20.9/F22/F23/F24/F25.0/F25.1/F25.8/F25.9/F28/F29/F30.10/F30.11/F30.12/F30.13/F30.2/F30.3/F30.4/F30.8/F30.9/F31.0/F31.10/F31.11/F31.12/F31.13/F31.2/F31.30/F31.31/F31.32/F31.4/F31.5/F31.60/F31.61/F31.62/F31.63/F31.64/F31.70/F31.71/F31.72/F31.73/F31.74/F31.75/F31.76/F31.77/F31.78/F31.81/F31.89/F31.9/F32.4/F32.5/F33.40/F33.41/F33.42/F34.8/F34.9/F39/F44.89/F84.3 |
| Depression | F32.0/F32.1/F32.2/F32.3/F32.8/F32.9/F33.0/F33.1/F33.2/F33.3/F33.8/F33.9/F34.1/F43.21 |
| Congestive heart failure | I09.81/I50 |
| Peripheral vascular disease | I70.0/I70.1/I70.201/I70.202/I70.203/I70.208/I70.209/I70.211/I70.212/I70.213/I70.218/I70.219/I70.221/I70.222/I70.223/I70.228/I70.229/I70.231/I70.232/I70.233/I70.234/I70.235/I70.238/I70.239/I70.241/I70.242/I70.243/I70.244/I70.245/I70.248/I70.249/I70.25/I70.261/I70.262/I70.263/I70.268/I70.269/I70.291/I70.292/I70.293/I70.298/I70.299/I70.301/I70.302/I70.303/I70.308/I70.309/I70.311/I70.312/I70.313/I70.318/I70.319/I70.321/I70.322/I70.323/I70.328/I70.329/I70.331/I70.332/I70.333/I70.334/I70.335/I70.338/I70.339/I70.341/I70.342/I70.343/I70.344/I70.345/I70.348/I70.349/I70.35/I70.361/I70.362/I70.363/I70.368/I70.369/I70.391/I70.392/I70.393/I70.398/I70.399/I70.401/I70.402/I70.403/I70.408/I70.409/I70.411/I70.412/I70.413/I70.418/I70.419/I70.421/I70.422/I70.423/I70.428/I70.429/I70.431/I70.432/I70.433/I70.434/I70.435/I70.438/I70.439/I70.441/I70.442/I70.443/I70.444/I70.445/I70.448/I70.449/I70.45/I70.461/I70.462/I70.463/I70.468/I70.469/I70.491/I70.492/I70.493/I70.498/I70.499/I70.501/I70.502/I70.503/I70.508/I70.509/I70.511/I70.512/I70.513/I70.518/I70.519/I70.521/I70.522/I70.523/I70.528/I70.529/I70.531/I70.532/I70.533/I70.534/I70.535/I70.538/I70.539/I70.541/I70.542/I70.543/I70.544/I70.545/I70.548/I70.549/I70.55/I70.561/I70.562/I70.563/I70.568/I70.569/I70.591/I70.592/I70.593/I70.598/I70.599/I70.601/I70.602/I70.603/I70.608/I70.609/I70.611/I70.612/I70.613/I70.618/I70.619/I70.621/I70.622/I70.623/I70.628/I70.629/I70.631/I70.632/I70.633/I70.634/I70.635/I70.638/I70.639/I70.641/I70.642/I70.643/I70.644/I70.645/I70.648/I70.649/I70.65/I70.661/I70.662/I70.663/I70.668/I70.669/I70.691/I70.692/I70.693/I70.698/I70.699/I70.701/I70.702/I70.703/I70.708/I70.709/I70.711/I70.712/I70.713/I70.718/I70.719/I70.721/I70.722/I70.723/I70.728/I70.729/I70.731/I70.732/I70.733/I70.734/I70.735/I70.738/I70.739/I70.741/I70.742/I70.743/I70.744/I70.745/I70.748/I70.749/I70.75/I70.761/I70.762/I70.763/I70.768/I70.769/I70.791/I70.792/I70.793/I70.798/I70.799/I70.8/I70.90/I70.91/I70.92/I71.00/I71.01/I71.02/I71.03/I71.1/I71.2/I71.3/I71.4/I71.5/I71.6/I71.8/I71.9/I72.0/I72.1/I72.2/I72.3/I72.4/I72.8/I72.9/I73.1/I73.81/I73.89/I73.9/I74.2/I74.3/I74.4/I76/I77.1/I77.71/I77.72/I77.73/I77.74/I77.79/I79.0/I79.1/I79.8/K55.1/K55.8/K55.9/Z95.820/Z95.828 |
| Valvular Heart Disease | A52.03/I05.0/I05.1/I05.2/I05.8/I05.9/I06.0/I06.1/I06.2/I06.8/I06.9/I07.0/I07.1/I07.2/I07.8/I07.9/I08.0/I08.1/I08.2/I08.3/I08.8/I08.9/I09.1/I09.89/I34.0/I34.1/I34.2/I34.8/I34.9/I35.0/I35.1/I35.2/I35.8/I35.9/I36.0/I36.1/I36.2/I36.8/I36.9/I37.0/I37.1/I37.2/I37.8/I37.9/I38/I39/Q23.0/Q23.1/Q23.2/Q23.3/Z95.2/Z95.3/Z95.4 |
| Hypertension | I10/O10.0/O10.9/I16/I67.4 |
| Chronic pulmonary disease | J40/J41.0/J41.1/J41.8/J42/J43.0/J43.1/J43.2/J43.8/J43.9/J44.0/J44.1/J44.9/J45.20/J45.21/J45.22/J45.30/J45.31/J45.32/J45.40/J45.41/J45.42/J45.50/J45.51/J45.52/J45.901/J45.902/J45.909/J45.990/J45.991/J45.998/J47.0/J47.1/J47.9/J60/J61/J62.0/J62.8/J63.0/J63.1/J63.2/J63.3/J63.4/J63.5/J63.6/J64/J65/J66.0/J66.1/J66.2/J66.8/J67.0/J67.1/J67.2/J67.3/J67.4/J67.5/J67.6/J67.7/J67.8/J67.9/J68.4 |
| Peptic ulcer disease | K25/K26/K27/K28 |
| Liver disease | K70.2/K70.3/K73/K71.7/K74.0/K74.2/K74.6/K74.3/K74.4/K74.5 |
| Renal Failure | N03/N05.2/N05.3/N05.4/N05.5/N05.6/N07.2/N07.3/N07.4/N01/N18/N19/N25 |
| Solid tumor without metastasis | C00.0/C00.1/C00.2/C00.3/C00.4/C00.5/C00.6/C00.8/C00.9/C01/C02.0/C02.1/C02.2/C02.3/C02.4/C02.8/C02.9/C03.0/C03.1/C03.9/C04.0/C04.1/C04.8/C04.9/C05.0/C05.1/C05.2/C05.8/C05.9/C06.0/C06.1/C06.2/C06.80/C06.89/C06.9/C07/C08.0/C08.1/C08.9/C09.0/C09.1/C09.8/C09.9/C10.0/C10.1/C10.2/C10.3/C10.4/C10.8/C10.9/C11.0/C11.1/C11.2/C11.3/C11.8/C11.9/C12/C13.0/C13.1/C13.2/C13.8/C13.9/C14.0/C14.2/C14.8/C15.3/C15.4/C15.5/C15.8/C15.9/C16.0/C16.1/C16.2/C16.3/C16.4/C16.5/C16.6/C16.8/C16.9/C17.0/C17.1/C17.2/C17.3/C17.8/C17.9/C18.0/C18.1/C18.2/C18.3/C18.4/C18.5/C18.6/C18.7/C18.8/C18.9/C19/C20/C21.0/C21.1/C21.2/C21.8/C22.0/C22.1/C22.2/C22.3/C22.4/C22.7/C22.8/C22.9/C23/C24.0/C24.1/C24.8/C24.9/C25.0/C25.1/C25.2/C25.3/C25.4/C25.7/C25.8/C25.9/C26.0/C26.1/C26.9/C30.0/C30.1/C31.0/C31.1/C31.2/C31.3/C31.8/C31.9/C32.0/C32.1/C32.2/C32.3/C32.8/C32.9/C33/C34.00/C34.01/C34.02/C34.10/C34.11/C34.12/C34.2/C34.30/C34.31/C34.32/C34.80/C34.81/C34.82/C34.90/C34.91/C34.92/C37/C38.0/C38.1/C38.2/C38.3/C38.4/C38.8/C39.0/C39.9/C40.00/C40.01/C40.02/C40.10/C40.11/C40.12/C40.20/C40.21/C40.22/C40.30/C40.31/C40.32/C40.80/C40.81/C40.82/C40.90/C40.91/C40.92/C41.0/C41.1/C41.2/C41.3/C41.4/C41.9/C43.0/C43.10/C43.11/C43.12/C43.20/C43.21/C43.22/C43.30/C43.31/C43.39/C43.4/C43.51/C43.52/C43.59/C43.60/C43.61/C43.62/C43.70/C43.71/C43.72/C43.8/C43.9/C45.0/C45.1/C45.2/C45.7/C47.0/C47.10/C47.11/C47.12/C47.20/C47.21/C47.22/C47.3/C47.4/C47.5/C47.6/C47.8/C47.9/C48.0/C48.1/C48.2/C48.8/C49.0/C49.10/C49.11/C49.12/C49.20/C49.21/C49.22/C49.3/C49.4/C49.5/C49.6/C49.8/C49.9/C4A0/C4A10/C4A11/C4A12/C4A20/C4A21/C4A22/C4A30/C4A31/C4A39/C4A4/C4A51/C4A52/C4A59/C4A60/C4A61/C4A62/C4A70/C4A71/C4A72/C4A8/C4A9/C50.011/C50.012/C50.019/C50.021/C50.022/C50.029/C50.111/C50.112/C50.119/C50.121/C50.122/C50.129/C50.211/C50.212/C50.219/C50.221/C50.222/C50.229/C50.311/C50.312/C50.319/C50.321/C50.322/C50.329/C50.411/C50.412/C50.419/C50.421/C50.422/C50.429/C50.511/C50.512/C50.519/C50.521/C50.522/C50.529/C50.611/C50.612/C50.619/C50.621/C50.622/C50.629/C50.811/C50.812/C50.819/C50.821/C50.822/C50.829/C50.911/C50.912/C50.919/C50.921/C50.922/C50.929/C51.0/C51.1/C51.2/C51.8/C51.9/C52/C53.0/C53.1/C53.8/C53.9/C54.0/C54.1/C54.2/C54.3/C54.8/C54.9/C55/C56.1/C56.2/C56.9/C57.00/C57.01/C57.02/C57.10/C57.11/C57.12/C57.20/C57.21/C57.22/C57.3/C57.4/C57.7/C57.8/C57.9/C58/C60.0/C60.1/C60.2/C60.8/C60.9/C61/C62.00/C62.01/C62.02/C62.10/C62.11/C62.12/C62.90/C62.91/C62.92/C63.00/C63.01/C63.02/C63.10/C63.11/C63.12/C63.2/C63.7/C63.8/C63.9/C64.1/C64.2/C64.9/C65.1/C65.2/C65.9/C66.1/C66.2/C66.9/C67.0/C67.1/C67.2/C67.3/C67.4/C67.5/C67.6/C67.7/C67.8/C67.9/C68.0/C68.1/C68.8/C68.9/C69.00/C69.01/C69.02/C69.10/C69.11/C69.12/C69.20/C69.21/C69.22/C69.30/C69.31/C69.32/C69.40/C69.41/C69.42/C69.50/C69.51/C69.52/C69.60/C69.61/C69.62/C69.80/C69.81/C69.82/C69.90/C69.91/C69.92/C70.0/C70.1/C70.9/C71.0/C71.1/C71.2/C71.3/C71.4/C71.5/C71.6/C71.7/C71.8/C71.9/C72.0/C72.1/C72.20/C72.21/C72.22/C72.30/C72.31/C72.32/C72.40/C72.41/C72.42/C72.50/C72.59/C72.9/C73/C74.00/C74.01/C74.02/C74.10/C74.11/C74.12/C74.90/C74.91/C74.92/C75.0/C75.1/C75.2/C75.3/C75.4/C75.5/C75.8/C75.9/C76.0/C76.1/C76.2/C76.3/C76.40/C76.41/C76.42/C76.50/C76.51/C76.52/C76.8/C7A.00/C7A.010/C7A.011/C7A.012/C7A.019/C7A.020/C7A.021/C7A.022/C7A.023/C7A.024/C7A.025/C7A.026/C7A.029/C7A.090/C7A.091/C7A.092/C7A.093/C7A.094/C7A.095/C7A.096/C7A.098/D03.0/D03.10/D03.11/D03.12/D03.20/D03.21/D03.22/D03.30/D03.39/D03.4/D03.51/D03.52/D03.59/D03.60/D03.61/D03.62/D03.70/D03.71/D03.72/D03.8/D03.9/E31.21/E31.22/E31.23 |
| Metastatic cancer | C77/C78/C79/C80 |
| Lymphoma | C81.00/C81.01/C81.02/C81.03/C81.04/C81.05/C81.06/C81.07/C81.08/C81.09/C81.10/C81.11/C81.12/C81.13/C81.14/C81.15/C81.16/C81.17/C81.18/C81.19/C81.20/C81.21/C81.22/C81.23/C81.24/C81.25/C81.26/C81.27/C81.28/C81.29/C81.30/C81.31/C81.32/C81.33/C81.34/C81.35/C81.36/C81.37/C81.38/C81.39/C81.40/C81.41/C81.42/C81.43/C81.44/C81.45/C81.46/C81.47/C81.48/C81.49/C81.70/C81.71/C81.72/C81.73/C81.74/C81.75/C81.76/C81.77/C81.78/C81.79/C81.90/C81.91/C81.92/C81.93/C81.94/C81.95/C81.96/C81.97/C81.98/C81.99/C82.00/C82.01/C82.02/C82.03/C82.04/C82.05/C82.06/C82.07/C82.08/C82.09/C82.10/C82.11/C82.12/C82.13/C82.14/C82.15/C82.16/C82.17/C82.18/C82.19/C82.20/C82.21/C82.22/C82.23/C82.24/C82.25/C82.26/C82.27/C82.28/C82.29/C82.30/C82.31/C82.32/C82.33/C82.34/C82.35/C82.36/C82.37/C82.38/C82.39/C82.40/C82.41/C82.42/C82.43/C82.44/C82.45/C82.46/C82.47/C82.48/C82.49/C82.50/C82.51/C82.52/C82.53/C82.54/C82.55/C82.56/C82.57/C82.58/C82.59/C82.60/C82.61/C82.62/C82.63/C82.64/C82.65/C82.66/C82.67/C82.68/C82.69/C82.80/C82.81/C82.82/C82.83/C82.84/C82.85/C82.86/C82.87/C82.88/C82.89/C82.90/C82.91/C82.92/C82.93/C82.94/C82.95/C82.96/C82.97/C82.98/C82.99/C83.00/C83.01/C83.02/C83.03/C83.04/C83.05/C83.06/C83.07/C83.08/C83.09/C83.10/C83.11/C83.12/C83.13/C83.14/C83.15/C83.16/C83.17/C83.18/C83.19/C83.30/C83.31/C83.32/C83.33/C83.34/C83.35/C83.36/C83.37/C83.38/C83.39/C83.50/C83.51/C83.52/C83.53/C83.54/C83.55/C83.56/C83.57/C83.58/C83.59/C83.70/C83.71/C83.72/C83.73/C83.74/C83.75/C83.76/C83.77/C83.78/C83.79/C83.80/C83.81/C83.82/C83.83/C83.84/C83.85/C83.86/C83.87/C83.88/C83.89/C83.90/C83.91/C83.92/C83.93/C83.94/C83.95/C83.96/C83.97/C83.98/C83.99/C84.00/C84.01/C84.02/C84.03/C84.04/C84.05/C84.06/C84.07/C84.08/C84.09/C84.10/C84.11/C84.12/C84.13/C84.14/C84.15/C84.16/C84.17/C84.18/C84.19/C84.40/C84.41/C84.42/C84.43/C84.44/C84.45/C84.46/C84.47/C84.48/C84.49/C84.60/C84.61/C84.62/C84.63/C84.64/C84.65/C84.66/C84.67/C84.68/C84.69/C84.70/C84.71/C84.72/C84.73/C84.74/C84.75/C84.76/C84.77/C84.78/C84.79/C84.90/C84.91/C84.92/C84.93/C84.94/C84.95/C84.96/C84.97/C84.98/C84.99/C84.A0/C84.A1/C84.A2/C84.A3/C84.A4/C84.A5/C84.A6/C84.A7/C84.A8/C84.A9/C84.Z0/C84.Z1/C84.Z2/C84.Z3/C84.Z4/C84.Z5/C84.Z6/C84.Z7/C84.Z8/C84.Z9/C85.10/C85.11/C85.12/C85.13/C85.14/C85.15/C85.16/C85.17/C85.18/C85.19/C85.20/C85.21/C85.22/C85.23/C85.24/C85.25/C85.26/C85.27/C85.28/C85.29/C85.80/C85.81/C85.82/C85.83/C85.84/C85.85/C85.86/C85.87/C85.88/C85.89/C85.90/C85.91/C85.92/C85.93/C85.94/C85.95/C85.96/C85.97/C85.98/C85.99/C86.0/C86.1/C86.2/C86.3/C86.4/C86.5/C86.6/C88.0/C88.2/C88.3/C88.4/C88.8/C88.9/C90.00/C90.01/C90.02/C90.10/C90.11/C90.12/C90.20/C90.21/C90.22/C90.30/C90.31/C90.32/C96.0/C96.2/C96.4/C96.9/C96.A/C96.Z/D47.Z9 |
| Coagulopathy | D65/D66/D67/D68.0/D68.1/D68.2/D68.311/D68.312/D68.318/D68.32/D68.4/D68.8/D68.9/D69.1/D69.3/D69.41/D69.42/D69.49/D69.51/D69.59/D69.6/D75.82/O99.111/O99.112/O99.113/O99.119/O99.12/O99.13 |
| Blood loss anemia | D50.0/O90.81/O99.011/O99.012/O99.013/O99.019/O99.02/O99.03 |
| Deficiency anemia | D50.1/D50.8/D50.9/D51.0/D51.1/D51.2/D51.3/D51.8/D51.9/D52.0/D52.1/D52.8/D52.9/D53.0/D53.1/D53.2/D53.8/D53.9/D63.0/D63.1/D63.8/D64.9 |
| Rheumatoid arthritis and collagen vascular disorders | L90.0/L94.0/L94.1/L94.3/M05.00/M05.011/M05.012/M05.019/M05.021/M05.022/M05.029/M05.031/M05.032/M05.039/M05.041/M05.042/M05.049/M05.051/M05.052/M05.059/M05.061/M05.062/M05.069/M05.071/M05.072/M05.079/M05.09/M05.10/M05.111/M05.112/M05.119/M05.121/M05.122/M05.129/M05.131/M05.132/M05.139/M05.141/M05.142/M05.149/M05.151/M05.152/M05.159/M05.161/M05.162/M05.169/M05.171/M05.172/M05.179/M05.19/M05.20/M05.211/M05.212/M05.219/M05.221/M05.222/M05.229/M05.231/M05.232/M05.239/M05.241/M05.242/M05.249/M05.251/M05.252/M05.259/M05.261/M05.262/M05.269/M05.271/M05.272/M05.279/M05.29/M05.30/M05.311/M05.312/M05.319/M05.321/M05.322/M05.329/M05.331/M05.332/M05.339/M05.341/M05.342/M05.349/M05.351/M05.352/M05.359/M05.361/M05.362/M05.369/M05.371/M05.372/M05.379/M05.39/M05.40/M05.411/M05.412/M05.419/M05.421/M05.422/M05.429/M05.431/M05.432/M05.439/M05.441/M05.442/M05.449/M05.451/M05.452/M05.459/M05.461/M05.462/M05.469/M05.471/M05.472/M05.479/M05.49/M05.50/M05.511/M05.512/M05.519/M05.521/M05.522/M05.529/M05.531/M05.532/M05.539/M05.541/M05.542/M05.549/M05.551/M05.552/M05.559/M05.561/M05.562/M05.569/M05.571/M05.572/M05.579/M05.59/M05.60/M05.611/M05.612/M05.619/M05.621/M05.622/M05.629/M05.631/M05.632/M05.639/M05.641/M05.642/M05.649/M05.651/M05.652/M05.659/M05.661/M05.662/M05.669/M05.671/M05.672/M05.679/M05.69/M05.70/M05.711/M05.712/M05.719/M05.721/M05.722/M05.729/M05.731/M05.732/M05.739/M05.741/M05.742/M05.749/M05.751/M05.752/M05.759/M05.761/M05.762/M05.769/M05.771/M05.772/M05.779/M05.79/M05.80/M05.811/M05.812/M05.819/M05.821/M05.822/M05.829/M05.831/M05.832/M05.839/M05.841/M05.842/M05.849/M05.851/M05.852/M05.859/M05.861/M05.862/M05.869/M05.871/M05.872/M05.879/M05.89/M05.9/M06.00/M06.011/M06.012/M06.019/M06.021/M06.022/M06.029/M06.031/M06.032/M06.039/M06.041/M06.042/M06.049/M06.051/M06.052/M06.059/M06.061/M06.062/M06.069/M06.071/M06.072/M06.079/M06.08/M06.09/M06.1/M06.20/M06.211/M06.212/M06.219/M06.221/M06.222/M06.229/M06.231/M06.232/M06.239/M06.241/M06.242/M06.249/M06.251/M06.252/M06.259/M06.261/M06.262/M06.269/M06.271/M06.272/M06.279/M06.28/M06.29/M06.30/M06.311/M06.312/M06.319/M06.321/M06.322/M06.329/M06.331/M06.332/M06.339/M06.341/M06.342/M06.349/M06.351/M06.352/M06.359/M06.361/M06.362/M06.369/M06.371/M06.372/M06.379/M06.38/M06.39/M06.4/M06.80/M06.811/M06.812/M06.819/M06.821/M06.822/M06.829/M06.831/M06.832/M06.839/M06.841/M06.842/M06.849/M06.851/M06.852/M06.859/M06.861/M06.862/M06.869/M06.871/M06.872/M06.879/M06.88/M06.89/M06.9/M08.00/M08.011/M08.012/M08.019/M08.021/M08.022/M08.029/M08.031/M08.032/M08.039/M08.041/M08.042/M08.049/M08.051/M08.052/M08.059/M08.061/M08.062/M08.069/M08.071/M08.072/M08.079/M08.08/M08.09/M08.1/M08.20/M08.211/M08.212/M08.219/M08.221/M08.222/M08.229/M08.231/M08.232/M08.239/M08.241/M08.242/M08.249/M08.251/M08.252/M08.259/M08.261/M08.262/M08.269/M08.271/M08.272/M08.279/M08.28/M08.29/M08.3/M08.40/M08.411/M08.412/M08.419/M08.421/M08.422/M08.429/M08.431/M08.432/M08.439/M08.441/M08.442/M08.449/M08.451/M08.452/M08.459/M08.461/M08.462/M08.469/M08.471/M08.472/M08.479/M08.48/M08.80/M08.811/M08.812/M08.819/M08.821/M08.822/M08.829/M08.831/M08.832/M08.839/M08.841/M08.842/M08.849/M08.851/M08.852/M08.859/M08.861/M08.862/M08.869/M08.871/M08.872/M08.879/M08.88/M08.89/M08.90/M08.911/M08.912/M08.919/M08.921/M08.922/M08.929/M08.931/M08.932/M08.939/M08.941/M08.942/M08.949/M08.951/M08.952/M08.959/M08.961/M08.962/M08.969/M08.971/M08.972/M08.979/M08.98/M08.99/M12.00/M12.011/M12.012/M12.019/M12.021/M12.022/M12.029/M12.031/M12.032/M12.039/M12.041/M12.042/M12.049/M12.051/M12.052/M12.059/M12.061/M12.062/M12.069/M12.071/M12.072/M12.079/M12.08/M12.09/M32.0/M32.10/M32.11/M32.12/M32.13/M32.14/M32.15/M32.19/M32.8/M32.9/M33.00/M33.01/M33.02/M33.09/M33.10/M33.11/M33.12/M33.19/M33.20/M33.21/M33.22/M33.29/M33.90/M33.91/M33.92/M33.99/M34.0/M34.1/M34.2/M34.81/M34.82/M34.83/M34.89/M34.9/M35.00/M35.01/M35.02/M35.03/M35.04/M35.09/M35.1/M35.3/M35.5/M35.8/M35.9/M36.0/M36.8/M45.0/M45.1/M45.2/M45.3/M45.4/M45.5/M45.6/M45.7/M45.8/M45.9/M46.00/M46.01/M46.02/M46.03/M46.04/M46.05/M46.06/M46.07/M46.08/M46.09/M46.1/M46.50/M46.51/M46.52/M46.53/M46.54/M46.55/M46.56/M46.57/M46.58/M46.59/M46.80/M46.81/M46.82/M46.83/M46.84/M46.85/M46.86/M46.87/M46.88/M46.89/M46.90/M46.91/M46.92/M46.93/M46.94/M46.95/M46.96/M46.97/M46.98/M46.99/M48.8X1/M48.8X2/M48.8X3/M48.8X4/M48.8X5/M48.8X6/M48.8X7/M48.8X8/M48.8X9/M49.80/M49.81/M49.82/M49.83/M49.84/M49.85/M49.86/M49.87/M49.88/M49.89 |
| Diabetes mellitus (uncomplicated) | E08.00/E08.01/E08.10/E08.11/E08.9/E09.00/E09.01/E09.10/E09.11/E09.9/E10.10/E10.11/E10.9/E11.00/E11.01/E11.9/E13.00/E13.01/E13.10/E13.11/E13.9/O24.011/O24.012/O24.013/O24.019/O24.02/O24.03/O24.111/O24.112/O24.113/O24.119/O24.12/O24.13/O24.311/O24.312/O24.313/O24.319/O24.32/O24.33/O24.811/O24.812/O24.813/O24.819/O24.82/O24.83/O24.911/O24.912/O24.913/O24.919/O24.92/O24.93 |
| Diabetes mellitus (complicated) | E08.21/E08.22/E08.29/E08.311/E08.319/E08.321/E08.329/E08.331/E08.339/E08.341/E08.349/E08.351/E08.359/E08.36/E08.39/E08.40/E08.41/E08.42/E08.43/E08.44/E08.49/E08.51/E08.52/E08.59/E08.610/E08.618/E08.620/E08.621/E08.622/E08.628/E08.630/E08.638/E08.641/E08.649/E08.65/E08.69/E08.8/E09.21/E09.22/E09.29/E09.311/E09.319/E09.321/E09.329/E09.331/E09.339/E09.341/E09.349/E09.351/E09.359/E09.36/E09.39/E09.40/E09.41/E09.42/E09.43/E09.44/E09.49/E09.51/E09.52/E09.59/E09.610/E09.618/E09.620/E09.621/E09.622/E09.628/E09.630/E09.638/E09.641/E09.649/E09.65/E09.69/E09.8/E10.21/E10.22/E10.29/E10.311/E10.319/E10.321/E10.329/E10.331/E10.339/E10.341/E10.349/E10.351/E10.359/E10.36/E10.39/E10.40/E10.41/E10.42/E10.43/E10.44/E10.49/E10.51/E10.52/E10.59/E10.610/E10.618/E10.620/E10.621/E10.622/E10.628/E10.630/E10.638/E10.641/E10.649/E10.65/E10.69/E10.8/E11.21/E11.22/E11.29/E11.311/E11.319/E11.321/E11.329/E11.331/E11.339/E11.341/E11.349/E11.351/E11.359/E11.36/E11.39/E11.40/E11.41/E11.42/E11.43/E11.44/E11.49/E11.51/E11.52/E11.59/E11.610/E11.618/E11.620/E11.621/E11.622/E11.628/E11.630/E11.638/E11.641/E11.649/E11.65/E11.69/E11.8/E13.21/E13.22/E13.29/E13.311/E13.319/E13.321/E13.329/E13.331/E13.339/E13.341/E13.349/E13.351/E13.359/E13.36/E13.39/E13.40/E13.41/E13.42/E13.43/E13.44/E13.49/E13.51/E13.52/E13.59/E13.610/E13.618/E13.620/E13.621/E13.622/E13.628/E13.630/E13.638/E13.641/E13.649/E13.65/E13.69/E13.8/P70.2 |
| Hypothyroidism | E00.0/E00.1/E00.2/E00.9/E01.8/E02/E03.0/E03.1/E03.2/E03.3/E03.8/E03.9/E89.0 |
| Obesity | E66.01/E66.09/E66.1/E66.2/E66.8/E66.9/O99.210/O99.211/O99.212/O99.213/O99.214/O99.215/R93.9/Z68.30/Z68.31/Z68.32/Z68.33/Z68.34/Z68.35/Z68.36/Z68.37/Z68.38/Z68.39/Z68.41/Z68.42/Z68.43/Z68.44/Z68.45/Z68.54 |
| Weight loss | E40/E41/E42/E43/E44.0/E44.1/E45/E46/E64.0/R63.4/R63.6 |
